# LncRNA GAS5 modulates the progression of non-small cell lung cancer through repressing miR-221-3p and up-regulating IRF2

**DOI:** 10.1186/s13000-021-01108-0

**Published:** 2021-05-22

**Authors:** Juan Ma, Haiyan Miao, Haiyun Zhang, Jingjing Ren, Shengyan Qu, Jing Da, Feifan Xu, Huan Zhao

**Affiliations:** 1 Department of Clinical Laboratory, The Sixth People’s Hospital of Nantong, Yonghe road No.500, 226011 Nantong, Jiangsu China; 2 Department of General Surgery, The Sixth People’s Hospital of Nantong, 226011 Nantong, Jiangsu China; 3 Department of Gastroenterology, The Sixth People’s Hospital of Nantong, 226011 Nantong, Jiangsu China; 4 Department of Respiration, The Sixth People’s Hospital of Nantong, Yonghe road No.500, 226011 Nantong, Jiangsu China

**Keywords:** NSCLC, lncRNA GAS5, miR-221-3p, IRF2

## Abstract

**Background:**

Long non-coding RNA growth arrest specific 5 (GAS5) is a regulator in non-small cell lung cancer (NSCLC) progression. Nonetheless, the mechanism by which GAS5 exerts its biological function in NSCLC cells remains unclear.

**Methods:**

GAS5, miR-221-3p relative expression levels in NSCLC tissues and cells were examined by qPCR. After gain-of-function and loss-of-function models were established, the viability of H1299 and A549 cells were examined by CCK-8 and EdU assays. Cell migration and invasion were examined by the Transwell experiment. The binding sequence of GAS5 for miR-221-3p was confirmed by the dual-luciferase reporter gene experiment. The regulatory function of GAS5 and miR-221-3p on IRF2 was investigated by Western blot.

**Results:**

GAS5 expression was down-modulated in NSCLC tissues and cell lines. GAS5 overexpression restrained the proliferation, migration and invasion of NSCLC cells, while miR-221-3p, which was targeted and negatively modulated by GAS5, worked oppositely. Restoration of miR-221-3p markedly reversed the effects of GAS5 on NSCLC cells. Additionally, GAS5 increased IRF2 expression in NSCLC cells by repressing miR-221-3p.

**Conclusions:**

GAS5 blocks the progression of NSCLC partly via increasing IRF2 expression level via repressing miR-221-3p.

## Introduction

Lung cancer is the most common cancer, with about 80 % of cases being non-small cell lung cancer (NSCLC) [1]. Despite recent advances in the diagnosis and treatment, the prognosis of patients with NSCLC is still unfavorable, with a 5-year overall survival rate of less than 20 % [2, 3]. Therefore, there is an urgent need for clarification of the mechanism of NSCLC progression, to develop novel treatment strategy for this disease.

Reportedly, long non-coding RNAs (lncRNAs) regulate the development of diverse cancers. It is reported that lncRNA growth arrest specific 5 (GAS5) is a tumor suppressor in multiple cancers including NSCLC [4, 5]. Nevertheless, the specific molecular mechanism by which GAS5 suppresses NSCLC progression has not been fully elucidated. MicroRNAs (miRNAs), are also vital regulators in diverse cancers. Accumulating studies indicate that miR-221-3p is a cancer-related miRNA. For instance, in cervical squamous cell carcinoma, miR-221-3p promotes angiogenesis and tumor growth by targeting THBS2 [6]; conversely, in ovarian cancer, miR-221-3p represses the proliferation and migration of cancer cells by targeting ARF4 [7]. Nonetheless, the role of miR-221-3p in NSCLC awaits further investigation.

Interferon regulatory factor 2 (IRF2) is a member of the interferon regulatory transcription factor (IRF) family. Reportedly, IRF2 is a direct target of miR-1290, and up-modulation of IRF2 can partially mitigate the promotion of NSCLC cell proliferation and invasion caused by miR-1290 overexpression, suggesting IRF2 is a tumor suppressor in NSCLC [8]. Nevertheless, the mechanism by which IRF2 is modulated in NSCLC is obscure.

In this work, we investigated the expression characteristics, biological functions of GAS5 and miR-221-3p in NSCLC, and we also studied the regulatory function of GAS5 on miR-221-3p. We proved that GAS5 repressed NSCLC progression via repressing miR-221-3p and up-regulating IRF2.

## Materials and methods

### Clinical specimens collection

Paracancerous and cancerous tissues (primary tumors) from a total of 58 NSCLC subjects from The Sixth People’s Hospital of Nantong were collected during surgery from October 2017 to October 2019. The research was endorsed by the Ethics Committee of the Sixth People’s Hospital of Nantong, and all patients signed an informed consent. None of the subjects received chemotherapy, radiotherapy or targeted therapy before the surgery. Tissues were preserved at -80 °C immediately after excision. The 58 subjects included 35 men and 23 women, 37 of whom were diagnosed with lung adenocarcinoma and 21 with lung squamous cell carcinoma.

### Cell culture and transfection

Human normal lung epithelial cell line BEAS-2B and NSCLC cell lines H1299, H1975, A549, and H2228 were available from American Type Culture Collection (ATCC) (Rockville, MD, USA). All cells were cultured in Dulbecco’s Modified Eagle’s Medium (DMEM) (Thermo Fisher Scientific, Waltham, MA, USA) containing 10 % fetal bovine serum (FBS) (Biyuntian, Shanghai, China) at 37 °C with 5 % CO_2_. 0.25 % trypsin (Biyuntian, Shanghai, China) was employed for subculture. The GAS5 overexpression plasmid, shRNA targeting GAS5, miR-221-3p mimics, and miR-221-3p inhibitors, and the corresponding controls were constructed by GenePharma (Shanghai, China). The above plasmids or oligonucleotides were transfected into H1299 or A549 cells using Lipofectamine®3000 (Invitrogen, Carlsbad, CA, USA).

### Quantitative real-time polymerase chain reaction (qPCR)

Total RNA of H1299 and A549 cells was extracted using TRIzol reagent (Thermo Fisher Scientific, Waltham, MA, USA), and RNA concentration, purity and integrity were measured. qPCR was performed using SYBR® Premix-Ex-Taq™ II kit (Takara, Tokyo, Japan). qPCR was conducted on ABI7500 FAST RT-PCR system (Thermo Fisher Scientific, Waltham, MA, USA). U6 was the internal reference for miR-221-3p, and glyceraldehyde 3-phosphate dehydrogenase (GAPDH) was the internal reference for GAS5 and IRF2. Relative expressions were quantified using the 2^−ΔΔCt^ method. The primer sequences were as follows:

miR-221-3p forward :5′-GAAGAAATGATTCCAGGTAGC-3′.

miR-221-3p reverse :5′-TGAACATCCAGGTCTGGGGCA-3′.

GAS5 forward:5’-TGGTTCTGCTCCTGGTAACG-3’.

GAS5 reverse:5’-AGGATAACAGGTCTGCCTGC-3’.

U6 forward:5′-TGCGGGTGCTCGCTTCGGCAGC-3′.

U6 reverse:5′-CCAGTGCAGGGTCCGAGGT-3′.

GAPDH forward:5′-GCACCGTCAAGGCTGAGAAC-3′.

GAPDH reverse:5′-TGGTGAAGACGCCAGTGGA-3′.

IRF2 forward: 5′-TGAAGTGGATAGTACG GTGAACA-3′.

IRF2 reverse: 5′-CGGATTGGTGACAA TCTCTTG-3′.

### Cell counting kit-8 (CCK-8) experiment

H1299 and A549 cells were seeded in 96-well plates (Corning, Corning, NY, USA) (2 × 10^3^ cells / well). Then the cell viability was assessed using CCK-8 (Dojindo, Tokyo, Japan) after 0, 24, 48, 72, and 96 h of culture, respectively. The absorbance of the cells at 450 nm was measured using a microplate reader (Molecular Devices, CA, USA).

### EdU experiment

When the confluency of H1299 and A549 cells reached about 60 %, 50 µmol/L EdU kit (Beyotime, Shanghai, China) was added to incubate the cells for 4 h. After that, the culture solution was discarded, and the cells were fixed in 4 % paraformaldehyde for 15 min, and then incubated with 0.2 % glycine for 10 min, and rinsed twice with PBS. Then the cells were incubated with 0.5 % Triton X-100, and Next, Apollo staining solution was added to incubate the cells for 30 min at room temperature in the dark. After that, DAPI staining solution was then added to mark the nuclei for 20 min at room temperature in the dark. Eventually, the cells were observed under a fluorescence microscope.

### Transwell experiment

Cell suspension of H1299 or A549 cells were prepared, and the cell density was adjusted to 1 × 10^5^ cells / mL in serum-free medium, and 100 µL of the cell suspension was added into the upper compartment of each Transwell insert (pore size, 8 μm; Corning, NY, USA). 100 µL of medium containing 10 % FBS was replenished into the lower compartment. Cells were cultured at 37 °C for 24 h, and then a cotton swab was used to remove the cells remaining in the upper compartment. Cells passing through the filter wells were fixed with 4 % paraformaldehyde, stained with crystal violet and observed under an inverted microscope (Olympus, Tokyo, Japan). When detecting the invasion of NSCLC cells, the basement membrane was covered with a layer of Matrigel (BD, San Diego, CA, USA) in advance, and the remaining experimental procedures were the same as for the migration assay.

### Dual-luciferase reporter gene experiment

Wild type (WT) / mutated (MUT) predicted binding sequences between miR-221-3p and GAS5, or between miR-221-3p and IRF2 3’-UTR were amplified and cloned into the pmirGLO Dual-Luciferase miRNA Target Expression Vector (Promega, Madison, WI, USA) to construct the luciferase reporter vectors. The above reporter vectors and miR-221-3p mimics, miR-221-3p inhibitors or their negative controls were co-transfected into H1299 or A549 cells, respectively, and the luciferase activity in each group was measured 48 h after transfection using the dual-luciferase assay system (Promega, Madison, WI, USA).

### Western blot

H1299 and A549 cells were lysed with RIPA buffer (Biosharp, Hefei, China) containing protease inhibitor PMSF (Biosharp, Hefei, China) to extract the total protein. Total protein concentration was determined by the BCA kit (Beyotime, Shanghai, China). Proteins were separated after sodium dodecyl sulfate-polyacrylamide gel electrophoresis and transferred to polyvinylidene fluoride (PVDF) membranes (Millipore, Bedford, MA, USA). The PVDF membranes were then blocked with 5 % skimmed milk, and incubated with primary antibody (anti-IRF2, ab124744, Abcam, Shanghai, China, 1:1000) overnight at 4 °C, and secondary antibodiy (ab150077, Abcam, Shanghai, China, 1:2000) for 1 h at room temperature, respectively. At last, Enhanced Chemiluminescence Western Blotting Substrate (Biozym, Hessisch Oldendorf, Germany) was utilized to develop the protein bands.

### Statistical analysis

All experiments were performed in triplicate. The data were statistically analyzed using SPSS 23.0 software (SPSS Inc., Chicago, IL, USA) and presented as mean ± SD. Student’s *t*-test was performed to evaluate the differences between the two groups. One-way ANOVA was applied for the comparisons of multiple groups. *P* < 0.05 signified statistical significance.

## Results

### GAS5 expression was abnormally decreased in NSCLC tissues and cell lines

**Fig. 1:**
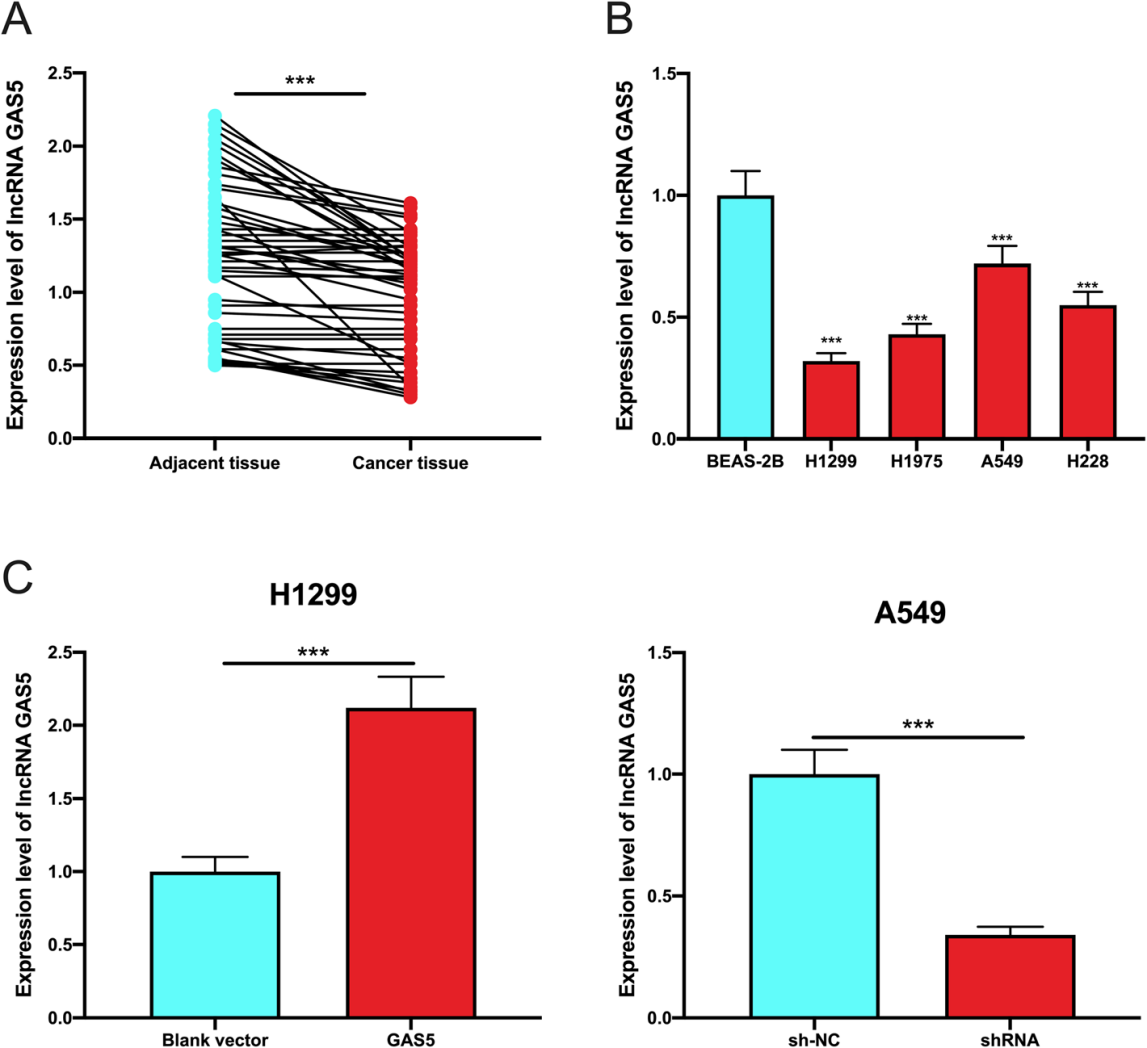
GAS5 expression in NSCLC. **a** GAS5 expression in NSCLC tissues and paracancerous tissues was detected using qPCR. **b** qPCR was utilized to detect GAS5 expression in normal lung epithelial cells and in each NSCLC cell line. **c** The transfection efficiency of GAS5 overexpression plasmid and shRNA was determined using PCR. *** represents *P* < 0.001

To detect GAS5 expression in NSCLC, firstly, qPCR was performed. As shown, GAS5 expression was reduced in both NSCLC tissues and cell lines compared with paracancerous tissues and normal lung cell BEAS-2B (Fig. 1 a, b). Among the NSCLC cell lines, GAS5 expression was the lowest in H1299 cells and the highest in A549 cells. Hence, the GAS5 overexpression plasmid was transfected into H1299 and the GAS5 shRNA was transfected into A549, respectively, to construct the gain-of-function and loss-of-function models, and the transfection efficiency was verified using qPCR (Fig. 1 c).

### GAS5 restrained the proliferation, migration and invasion of NSCLC cells

**Fig. 2:**
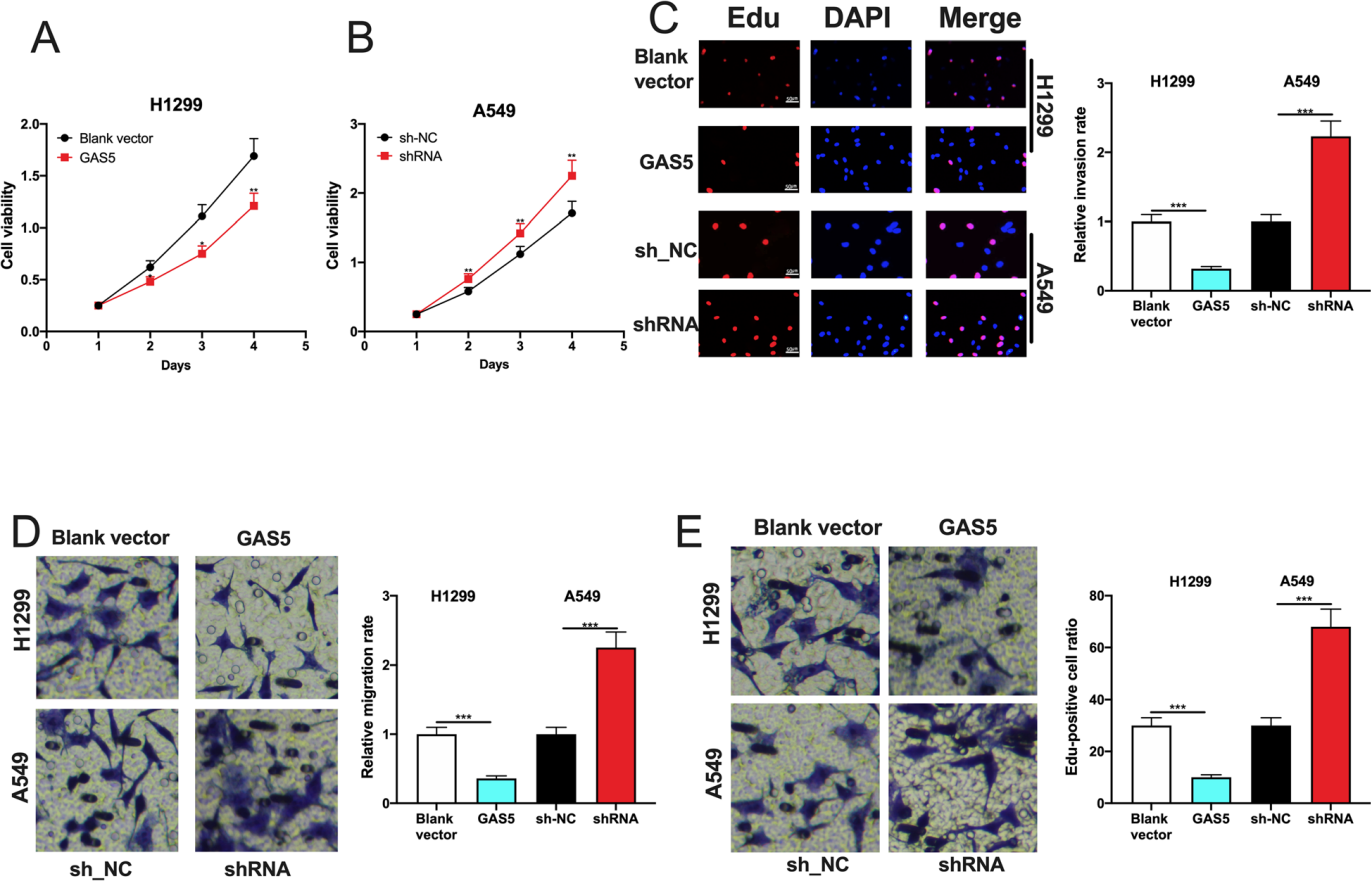
Effects of GAS5 on NSCLC cells. **a**, **b** CCK-8 was executed to detect the effects of overexpression and knockdown of GAS5 on the viability of H1299 and A549 cells, respectively. **c** EdU experiment was executed to detect the effects of overexpression and knockdown of GAS5 on the proliferation of H1299 and A549 cells, respectively. **d**, **e** Transwell was used to analyze the effects of overexpression and knockdown of GAS5 on the migration and invasion of H1299 and A549 cells, respectively. *, **, *** represent *P* < 0.05, *P* < 0.01, *P* < 0.001

To investigate the effect of GAS5 dysregulation on NSCLC cells, CCK-8 experiments were executed. GAS5 overexpression in H1299 cells was uncovered to markedly impeded cell viability, and transfection of GAS5 shRNA remarkably enhanced the cell viability of A549 cells (Fig. 2 a, b). The results of EdU experiments indicated that GAS5 overexpression remarkably repressed the proliferation of NSCLC cells, while knocking down GAS5 exerted the opposite effect on A549 cells (Fig. 2 c). Additionally, GAS5 overexpression remarkably restrained the migration and invasion of H1299 cells, and GAS5 knockdown markedly promoted the migration and invasion of A549 cells (Fig. 2 d, e).

### miR-221-3p was a downstream target of GAS5

**Fig. 3:**
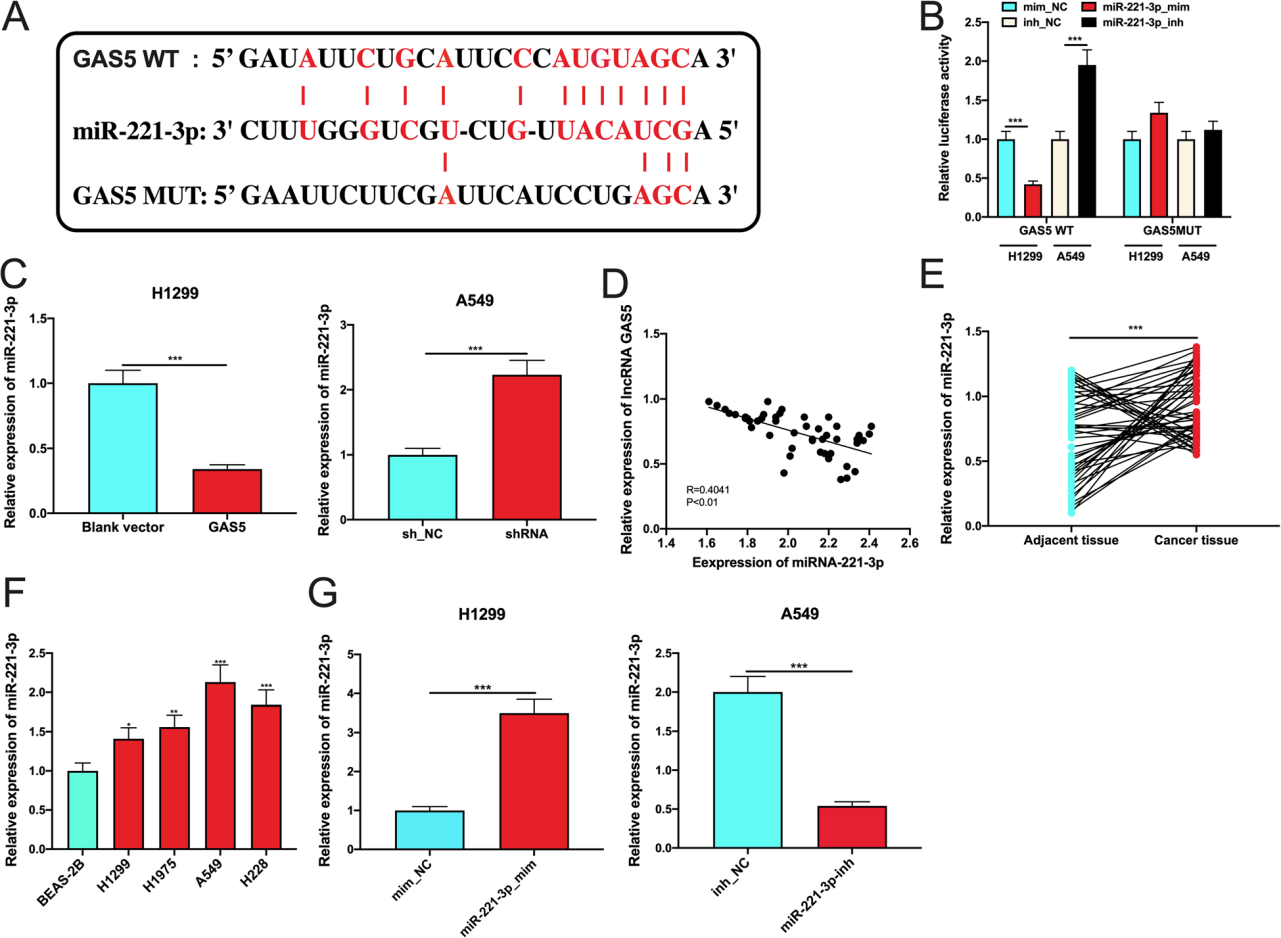
miR-221-3p was validated to be one of the downstream targets of GAS5. **a** The binding sequence between GAS5 and miR-221-3p was predicted by the StarBase database. **b** Verification of the binding site between miR-221-3p and GAS5 was conducted by dual-luciferase reporter gene experiment. **c** The effects of overexpression and knockdown of GAS5 on miR-221-3p expression in NSCLC cells were detected by qPCR. **d** The correlation between GAS5 and miR-221-3p expressions in NSCLC tissues. **e** MiR-221-3p expression in NSCLC tissues and paracancerous tissues was detected by qPCR. **f** MiR-221-3p expression in human normal lung epithelial cell line and each NSCLC cell line was detected by qPCR. **g** Transfection efficiency of miR-221-3p mimics and miR-221-3p inhibitors was verified using PCR. *** represents *P* < 0.001

The ENCORI database (http://starbase.sysu.edu.cn/) predicted that miR-221-3p was one of the direct downstream targets of the GAS5 (Fig. 3 a). In dual-luciferase reporter gene experiments, miR-221-3p mimics repressed the luciferase activity of wild type GAS5 reporter, and miR-221-3p inhibitors promoted that; however, the selective regulation of miR-221-3p had no effects on the luciferase activity of mutated GAS5 reporter, which confirmed that miR-221-3p could bind with GAS5 (Fig. 3 b). From qPCR, it was observed that miR-221-3p expression was remarkably repressed by GAS5 overexpression in H1299 cells, but knockdown of GAS5 facilitated miR-221-3p expression in A549 cells (Fig. 3 c). Additionally, GAS5 expression and miR-221-3p expression showed a negative correlation in NSCLC specimens (Fig. 3d). Furthermore, in contrast to GAS5, miR-221-3p expression was markedly enhanced in NSCLC tissues and cell lines (Fig. 3e, f). Therefore, we hypothesized that GAS5 may regulate the phenotypes of NSCLC cells through miR-221-3p. Because miR-221-3p expression was the lowest in A549 cells and the highest in H1299 cells, we transfected miR-221-3p mimics and miR-221-3p inhibitors into A549 and H1299 cells, respectively, to construct gain-of-function and loss-of-function models (Fig. 3g).

### miR-221-3p enhanced the proliferation, migration and invasion of NSCLC cells

**Fig. 4:**
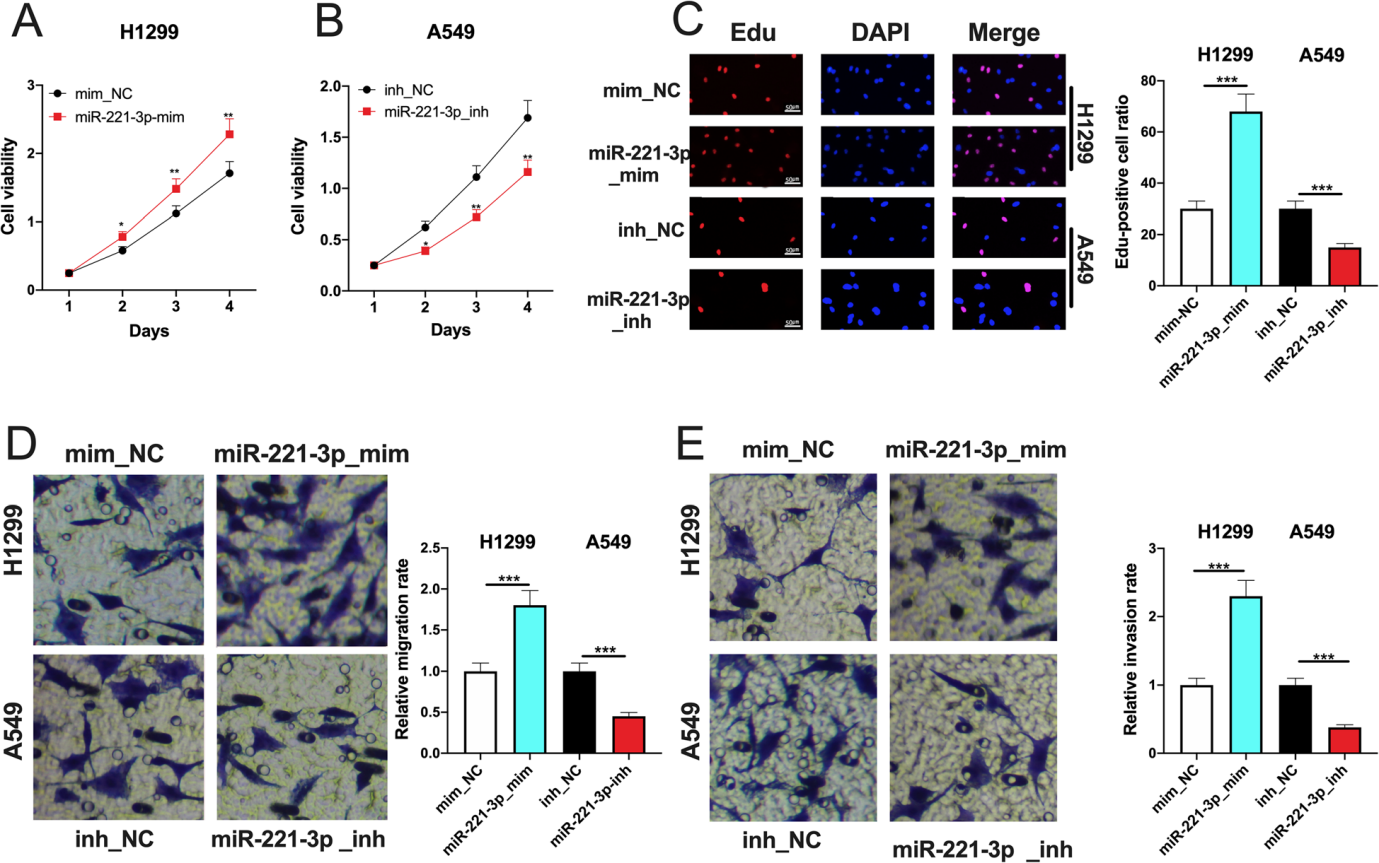
Effects of miR-221-3p on NSCLC cells. **a**, **b** CCK-8 experiment was adopted to detect the effects of overexpression or inhibition of miR-221-3p on the viability of A549 and H1299 cells, respectively. **c** EdU experiment was applied to detect the effect of overexpression or inhibition of miR-221-3p on the proliferation of A549 and H1299 cells, respectively. **d**, **e** Transwell expeimrnt was employed to detect the effects of overexpression or inhibition of miR-221-3p on the migration and invasion of A549 and H1299 cells, respectively. *, **, *** represent *p* < 0.05, *p* < 0.01, and *p* < 0.001, respectively

CCK-8 and EdU experiments confirmed that miR-221-3p overexpression in A549 remarkably enhanced the proliferation of A549 cells (Fig. 4 a-c). In Transwell experiments, migration and invasion of A549 cells transfected with miR-221-3p mimics were markedly enhanced (Fig. 4d, e). Conversely, the proliferation, migration and invasion of H1299 cells transfected with the miR-221-3p inhibitors were significantly suppressed (Fig. 4 a-e). These results indicated that miR-221-3p was an oncomiR.

### miR-221-3p counteracted the effect of GAS5 on NSCLC cells

**Fig. 5:**
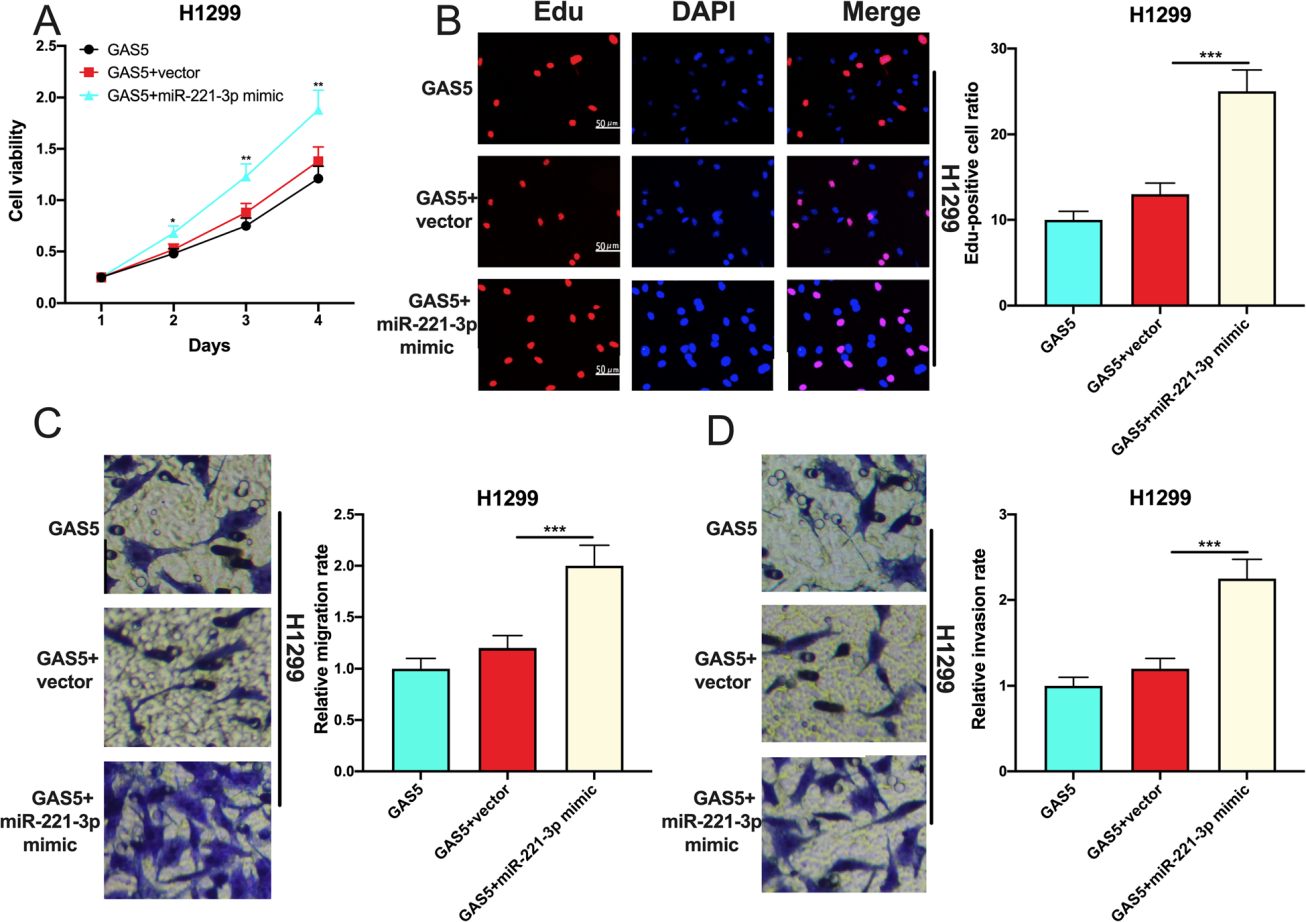
MiR-221-3p counteracted the biological functions of GAS5 on NSCLC cells. **a** The effects of co-transfection of GAS5 overexpression plasmid and miR-221-3p mimics on H1299 cell viability were detected by CCK-8 experiment. **b** The effects of co-transfection of GAS5 overexpression plasmid and miR-221-3p mimics on the proliferation of H1299 cells were detected by EdU assay. **c**, **d** Transwell experiment was conducted to detect the effects of co-transfection of GAS5 overexpression plasmid and miR-221-3p mimics on H1299 cell. *, **, *** represent *p* < 0.05, *p* < 0.01, and *p* < 0.001, respectively

We then co-transfected miR-221-3p mimics and GAS5 overexpression plasmids into H1299 cells. CCK-8 and EdU experiments indicated that miR-221-3p mimics could counteract the effect of GAS5 on suppressing H1299 cell viability and proliferation (Fig. 5 a, b). Moreover, the effects of GAS5 on repressing the migration and invasion of H1299 cells was also reversed by miR-221-3p overexpression (Fig. 5 c, d). The above data implied that, to some extent, GAS5 exerted its tumor-suppressive effects by specifically repressing miR-221-3p.

### GAS5 indirectly modulated IRF2 via miR-221-3p

**Fig. 6:**
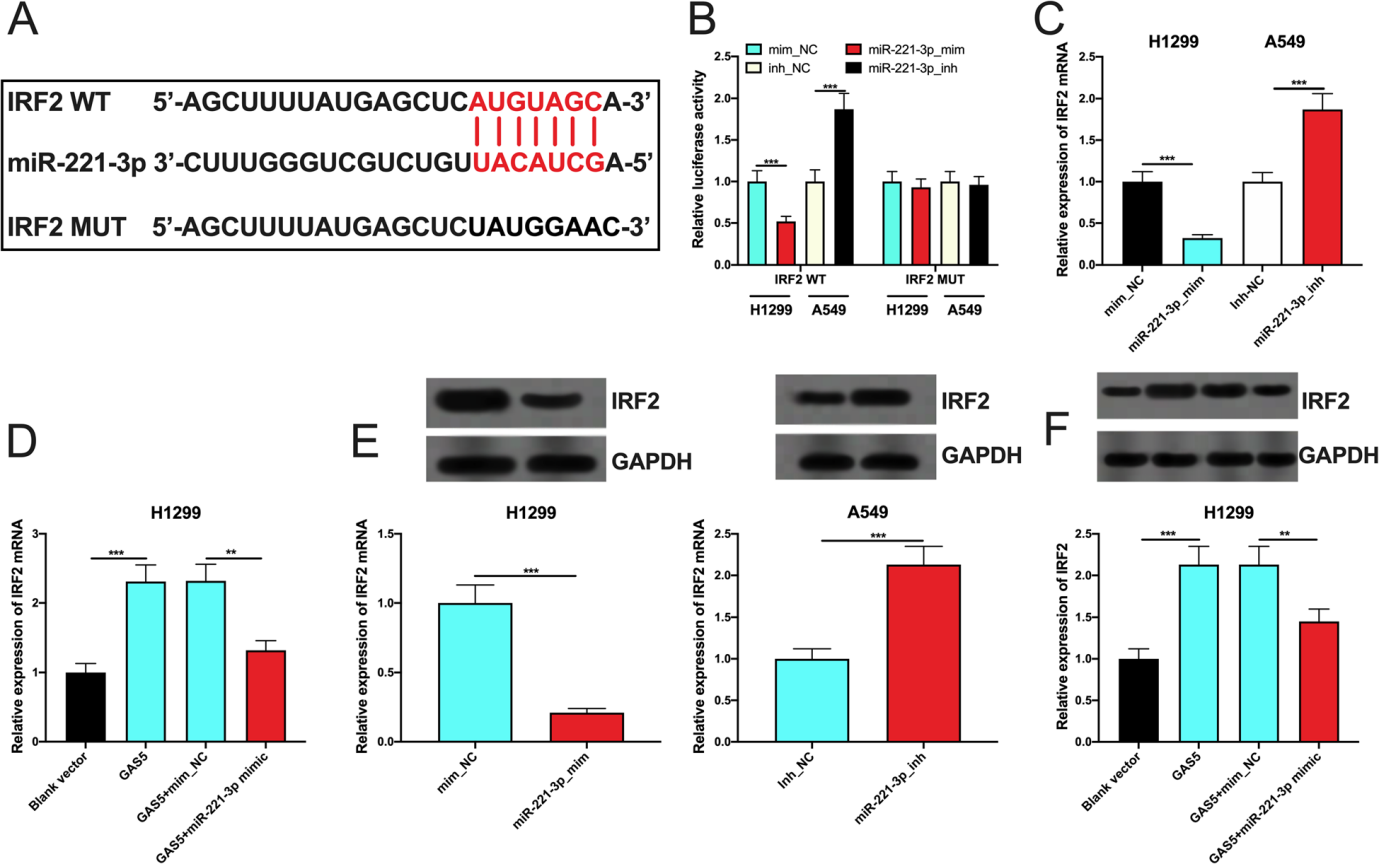
Regulatory effects of GAS5 and miR-221-3 on IRF2 expression in NSCLC cells. **a** The binding sequence between miR-221-3p and IRF2 3’UTR. **b** Binding sites between miR-221-3p and IRF2 3’UTR were verified using dual-luciferase reporter gene experiments. **c**, **d** The regulatory effects of miR-221-3p and GAS5 on IRF2 mRNA expression in NSCLC cells were detected by qPCR. **e**, **f** The effects of miR-221-3 and GAS5 on IRF2 protein expression in NSCLC cells were detected by Western blot. ** and *** represent *p* < 0.01 and *p* < 0.001, respectively

Next, miRNAwalk and miRNAmap databases were used to co-predict the downstream target genes of miR-221-3p, and IRF2 was noticed (Fig. 6 a). Additionally, IRF2 is reported to be a target gene of miR-221-3p [9]. In dual-luciferase reporter experiment, it was observed that the luciferase activity of the wild type IRF2 reporter was markedly repressed by miR-221-3p mimics and significantly promoted by miR-221-3p inhibitors, but no significant changes were observed in the luciferase activity of mutated IRF2 reporter vector (Fig. 6b). qPCR and Western blot experiments indicated that miR-221-3p could inhibit IRF2 expression at both miRNA and protein levels, while GAS5 exerted the opposite effects, and miR-221-3p could reverse the effect of GAS5 (Fig. 6 c-f). With these data, we could conclude that GAS5 indirectly enhanced IRF2 expression in NSCLC cells through miR-221-3p.

## Discussion

NSCLC is one of the most deadly cancers. In recent years, more and more lncRNAs have been found to be involved in regulation of tumorigenesis and cancer development [10]. Reportedly, GAS5 is abnormally expressed in leukemia, cervical cancer, breast cancer, ovarian cancer, prostate cancer, bladder cancer, gastric cancer, colorectal cancer, liver cancer, osteosarcoma, glioma and lung cancer, and it exerts a tumor-suppressive effect in cancers [11, 12]. For instance, in colorectal cancer, GAS5 blocks disease progression through inducing YAP phosphorylation and degradation [13]. In NSCLC, GAS5 also shows the potential to be a biomarker and therapy target. Receiver Operating Characteristic (ROC) curve analysis suggests that GAS5 in exosomes in combination with CEA is an ideal non-invasive diagnostic strategy for identifying patients with early NSCLC [14]. Another study reports that, knocking down GAS5 in NSCLC cells can improve the viability of NSCLC cells and increase their resistance to cisplatin [15]. Additionally, a recent study demonstrates that GAS5 overexpression sensitizes A549 cells to radiotherapy through regulating miR-21/PTEN/Akt axis [16]. Another study reports that, GAS5 represses lung cancer cell proliferation and metastasis via regulating miR-205/PTEN axis [5]. In the present study, we also demonstrated that GAS5 was down-regulated in NSCLC tissues and cell lines, and functional experiments suggested that GAS5 suppressed the proliferation, migration and invasion of H1299 and A549 cell lines, showing properties of a tumor-suppressive lncRNA, which is consistent with previous reports [5, 15, 16]. It is well known that lncRNA can function as ceRNA to regulate the expression of multiple miRNAs. It has been confirmed that GAS5 can sponge miR-205 and regulate the expression of PTEN [5]. In this study, we find that miR-221-3p is also a target of GAS5.

MiR-221-3p is reported to show cancer-promoting effects [17]. For instance, it has been found that miR-221-3p expression is remarkably up-modulated in hepatoma tissues and cells, and it facilitates cancer cell proliferation and migration through repressing Axin2 [18]. In pancreatic cancer tissues, miR-221-3p expression is discovered to be remarkably increased, and the ROC curve indicates that it has a better specificity for the diagnosis of distant metastasis, compared with CA19-9 [19]. A recent study reports that, miR-221-3p promotes the growth of NSCLC cells by targeting p27 [20]. In the present work, to probe the downstream mechanism of GAS5 in NSCLC, miR-221-3p was predicted and verified as one of the direct downstream targets of GAS5, which could be negatively regulated by GAS5. This regulatory relationship is consistent with previous reports [21, 22]. In breast cancer, miR-221 is the target of GAS5 [21], and GAS5 regulates DKK2 expression by competitively binding miR-221-3p, inhibiting the activation of Wnt / β-Catenin pathway, enhancing the anti-tumor effect of adriamycin [22]. We demonstrated that miR-221-5p was highly expressed in NSCLC, and it facilitated the malignant phenotypes of NSCLC cells; additionally, miR-221-3p over-expression reversed the effects of GAS5 on NSCLC cells. Based on these results, we confirmed that miR-221-5p was an oncomiR in NSCLC, and GAS5 suppressed NSCLC progression by repressing miR-221-3p.

IRF2 is reported to be a tumor suppressor in some cancers. In liver cancer, IRF2 can inactivate the STAT3 signaling pathway [23]. In gastric cancer, IRF2 can positively regulate p53, the famous tumor suppressor [24]. What’s more, a recent study reports that loss of IRF2 leads to immune evasion of cancer cells through repressing MHC Class I antigen presentation and increasing PD-L1 expression [25]. In NSCLC, IRF2 is reported to be underexpressed, and it has been identified as the target gene of miR-1290 and miR-18a-5p [8, 26]. In the present study, IRF2 was verified as a novel target gene of miR-221-3p in NSCLC. Additionally, we demonstrated that GAS5 could positively regulate IRF2 expression via repressing miR-221-3p. Competitive endogenous RNA (ceRNA) mechanism is a classical approach by which lncRNA exerts its biological functions. Collectively, our results depict a novel ceRNA network consisting of GAS5, miR-221-3p and IRF2, which can regulate NSCLC progression.

There are some limitations in this work. Firstly, there are only *in vitro* experiments in the present study, and *in vivo* data are essential to consolidate our conclusion. Secondly, the regulatory functions of GAS5 on other phenotypes of NSCLC cells, such as cell cycle progression, apoptosis, chemoresistance, and immune evasion, awaits further investigation. Last but not least, the relationship between GAS5/miR-221-3p/IRF2 and NSCLC patients’ prognosis has not been explored, and this is crucial to evaluate the potential of these molecules as biomarkers.

## Conclusions

In summary, through a series of *in vitro* experiments, we conclude that GAS5 can target and repress miR-221-3p, thereby modulating IRF2 expression and exerts a role in blocking NSCLC progression. This work lays a theoretical foundation for the diagnosis and treatment of NSCLC.

## Data Availability

The data used to support findings of this study are available from the corresponding author upon request.

## Abbreviations

NSCLC: Non-small cell lung cancer
GAS5: Growth arrest specific 5
IRF2: Interferon regulatory factor 2
qRT-PCR: Quantitative reverse transcription polymerase chain reaction
DMEM: Dulbecco’s Modified Eagle’s Medium
FBS: Fetal bovine serum
GAPDH: Glyceraldehyde 3-phosphate dehydrogenase
